# Re: Efficacy and Safety of Vildagliptin and Remogliflozin as Add-on Therapy to Metformin in Patients of Type 2 Diabetes Mellitus

**DOI:** 10.18295/squmj.12.2023.092

**Published:** 2024-08-29

**Authors:** Hunain Raza, Muhammad E. Bhutta, Muhammad H. Siddique

**Affiliations:** Islamic International Medical College, Riphah International University, Rawalpindi, Pakistan

Dear Editor,

I read with interest the original study by Sharma *et al*. published in the May 2024 issue of SQUMJ which aimed to investigate the efficacy and safety of remogliflozin, a novel sodium-glucose cotransporter subtype-2 (SGLT2) inhibitor, and vildagliptin, a commonly prescribed dipeptidyl peptidase-4 inhibitor as an add-on therapy to metformin in type 2 diabetes mellitus (T2DM) management.^1^ T2DM is a common chronic illness linked to rising rates of morbidity and mortality. In 2010, the global prevalence of diabetes among adults was approximately 6.4%, affecting 285 million people and predicted to increase to 7.7% by 2030, affecting over 439 million people worldwide.^2^

Sharma *et al*.’s prospective, open-label, parallel-group, interventional, and comparative study recruited 60 T2DM aged 35–70 years with glycated haemoglobin (HbA1c) >6.5% taking metformin on a daily dose of 1,500–3,000 mg for ≥3 months and randomised them into 2 groups that received either vildagliptin (50 mg) or remogliflozin (100 mg) twice daily for 90 days. The group that received remogliflozin showed significant improvement in HbA1c levels compared to the vildagliptin group after 90 days of treatment (−0.67 ± 0.24 versus −0.20 ± 0.22%; *P* <0.001). Weight loss was also significantly more in the remogliflozin group than in the vildagliptin group relative to the baseline values (−3.73 ± 1.91 versus −0.4 ± 1.52 kg; *P* <0.01).^1^ The remogliflozin group showed statistically significant reductions in total cholesterol, triglycerides, low-density lipoprotein (LDL), and very LDL (VLDL) compared to the vildagliptin group. Additionally, the increase in high-density lipoprotein levels was also significantly greater in the remogliflozin group. The adverse effects reported in both cases were mild and self-limiting, resolving spontaneously during the study period. Notably, neither group experienced any episode of hypoglycaemia, suggesting that both drugs are suitable for hypoglycaemia-prone patients.

The trial results are promising and the study’s contribution to advancing diabetes management by exploring novel treatment options and demonstrating the superior efficacy of remogliflozin over vildagliptin in glycaemic control and weight loss is highly commendable. However, several limitations impact the generalisability of the findings such as sample size, short follow-up duration and recruitment of patients from a specific ethnic group.

While the study demonstrated a statistically significant decrease in mean cholesterol levels, mean LDL levels and mean VLDL levels with remogliflozin compared to vildagliptin, these findings are contrast to the findings of a previous study.^3^ Sykes *et al*. included a larger sample size and showed increments in total cholesterol, LDL and VLDL levels by 2.5%, 4.9% and 1.2%, respectively.^3^ Therefore, it is suggested that future research on this drug be large-scale, to determine whether it has lipid-lowering potential or not.

Future research should focus on long-term observations assessing the treatment impact on renal and cardiovascular outcomes in T2DM patients and should also investigate various drugs within the SGLT2 inhibitors class to identify the optimal add-on drug for T2DM patients. Zinman *et al*. showed that T2DM patients at high risk for cardiovascular events who received empagliflozin, compared with a placebo, had a lower rate of the primary composite cardiovascular outcome and death from any cause when the study drug was added to standard care.^4^

Newer treatment options such as dual glucose-dependant insulinotropic polypeptide and glucagon-like peptide-1 receptor agonists such as tirzepatide should be evaluated and adopted. Clinical trials have demonstrated that tirzepatide significantly reduces HbA1c levels and induces substantial weight loss, as well as reductions in parameters commonly associated with heightened cardiometabolic risk such as blood pressure, visceral adiposity and circulating triglycerides, addressing key challenges in T2DM management.^5^

Sharma *et al*.’s thorough assessment of remogliflozin and vildagliptin as adjuvant medicines in the management of T2DM has greatly advanced our knowledge of available treatments for individuals with inadequate glycaemic control.^1^ The study highlights the need for continued research to optimise treatment regimens for patients with T2DM and establishes a solid foundation for improving diabetes care.
